# Readmission After Geriatric Inpatient Care: A Narrative Review and a Comparative Analysis

**DOI:** 10.1177/21501319251320181

**Published:** 2025-02-27

**Authors:** Carl Willers, Rikard Lindqvist, Martin Dreilich, Stefan Fors, Amelie Lindh Mazya, Gunnar H. Nilsson, Anne-Marie Boström, Mahwish Naseer, Elisabeth Rydwik

**Affiliations:** 1 Karolinska Institutet, Huddinge, Sweden; 2 FOU nu, Research and Development Center for the Elderly, Järfälla, Sweden; 3 ASIH Capio, Stockholm, Sweden; 4 Karolinska Institutet & Stockholm University, Stockholm, Sweden; 5 Region Stockholm, Stockholm, Sweden; 6 Geriatric Department of Danderyd Hospital, Stockholm, Sweden; 7 Academic Primary Care Center, Stockholm, Sweden; 8 Karolinska University Hospital, Huddinge, Sweden; 9 Stockholms Sjukhem, Stockholm, Sweden; 10 Karolinska University Hospital, Solna, Sweden

**Keywords:** care transition, geriatric care, post-discharge care, older adults, readmission, narrative review

## Abstract

**Background:**

Readmission can be be related to the work of several stakeholders involved in the care of individuals throughout the community, including, for example, primary care and social care providers. A narrative review was performed to assess definitions and frequency of readmission for older adults found in previous research. In addition, a dataset for a cohort of older adults in Stockholm, Sweden, was used to quantify how different definitions of readmission affect frequency.

**Materials and Methods:**

The review was based on pre-specified search criteria within PubMed and Embase databases. All studies based on a cohort of older adults with a primary objective to assess readmission to inpatient care, were included for the assessment of readmission criteria. The dataset was based on a cohort of older adults treated at a geriatric department in Stockholm during 2016. Estimations of readmission were performed with the most common criteria found in the narrative review.

**Results:**

The narrative review showed that definitions of readmission included predominantly time-based criteria, either alone or combined with additional criteria such as medical condition or readmitting department. Frequency of readmission based on different definitions varied substantially; a 14-day time interval implied a rate of 8.0% whilst a 30-day interval—more commonly used—rendered a rate of 12.6%. The density of readmissions per day was higher during the first weeks after discharge, and then dropped continuously.

**Conclusion:**

Transparency on definitions is imperative in studies that include rates of readmission. The levels of readmission rates are highly dependent on the study population and its context. Furthermore, the actual value of readmission monitoring is dependent on what purpose it is supposed to fulfill, and it is essential to put it into context of all relevant stakeholders including, for example, the primary care providers and different social care providers throughout the community.

## Introduction

Studies of hospital readmission are common, and readmission rate continuously recurs as a measure for quality of care.^1^ There are government initiatives in countries such as France^2^ and Sweden^3^ with focus on assessing burden of readmission. There are also several cases where readmission has been used not only as an indicator of quality of care delivered but also as basis for reimbursement adjustments.^4^ In the United States for example, financial penalties aimed also at skilled nursing facilities have been designed.^5^ Research on effects of such penalty programs show possible effects in terms of reduced risk of readmission.^6^ Regardless the purpose, the concept of readmission is dependent on several stakeholders within the health system—including the inpatient care facility, the primary care providers, and other providers of social care throughout the community such as home help and residential care—and should be managed as such.^7^ It is not controversial to state that post-discharge care may have an important impact on future health and care service utilization.^8^

Geriatric medicine and its organization vary between countries, and levels of available resources allocated may vary also between a country’s regions. There are initiatives focusing on establishing international guidelines and developing geriatric medicine as an independent specialty, such as European Geriatric Medicine Society.^9^ Since 1969 geriatrics is a recognized specialty in Sweden, and the care often includes short-term rehabilitation after inpatient care.^10^ The responsibility of inpatient and outpatient healthcare is regional, whereas the municipality finances healthcare for older adults living at home and social care including home-care services.^11^

Readmission is of particular interest for this vulnerable group of individuals given its limited ability to navigate the complex healthcare system.^12^ One particularly critical moment in the continuum of care for older adults is the transition between different responsible authorities, between care financed and delivered by the region and by the municipality. This often takes place immediately after discharge from inpatient geriatric care and readmission may be considered a result of low-quality coordination. Here, previous research has shown that the primary care provider has an important role to play^13^ together with the providers of social home care.^14^ Other studies highlight that insufficient volume or competence in the delivery of care, can increase the risk of readmission.^15,16^ Furthermore, there are studies assessing older adults’ readmission risk, although mostly related to particular interventions or conditions.^17^

There is ambiguity regarding how to define a readmission, except the fact that it comes chronologically in time after the index admission. Limited research has been done on how different definitions and criteria impact the frequency of readmission. It would therefore be of relevance to explore criteria’s importance for the frequency of readmission—especially since the definitions of readmission may have implications that go far beyond the scope of a single study, for example, computation of the monetary burden of disease, resource allocation to primary care, or cost effectiveness of an intervention.

The objectives of the present study were:

1. to assess
  a. definitions of readmission in previous studies of older adults,
  b. frequency of inpatient readmission in previous studies of geriatric care, and
2. to analyze how different definitions identified may impact the frequency of readmission.

## Materials and Methods

### Design

The present study entails a narrative review of available research regarding readmission of older adults, with aims to assess definitions and frequency of readmission. The study also includes a comparative quantification of readmission rates with the aim to assess how different definitions affect frequency, based on definitions identified via the narrative review, in a cohort of patients described in previous studies.^18,19^ The current cohort has also been subject to more extensive analysis on primary care utilization and its possible association to readmission,^20^ as well as readmission in relation to degree of social home care via the municipality.^14^

### Narrative Review

#### Search strategy and inclusion/exclusion criteria

A narrative review was performed based on pre-specified search criteria developed together with research literature expertise at the Karolinska Institutet University Library. The initial search of published literature was conducted within the PubMed and Embase databases. The search was restricted to include only research literature written in English and published between Jan 1st 2000 and Dec 31st 2022. All studies based on a cohort of older adults with a primary objective to assess readmission to inpatient care, were included for the assessment of readmission criteria. The selection was performed by one research group member (CW) and validated by one of two others (ER and MN); if any deviations were identified during validation, these were iterated until agreement was reached. The search criteria are available in Supplemental Table S1 and the search results are presented in Supplemental Figure S1 in accordance with the PRISMA process.^21^

#### Definitions of readmission

The first part of aim 1 was to assess definitions of readmission. After selection of studies that evaluated the rate of inpatient readmission for older adults as a primary objective (Supplemental Figure S1), the definitions used across the studies were identified and categorized according to type (eg, time-based and diagnosis-based).

#### Frequency of readmission

The second part of aim 1 was to assess frequency of readmission. To enable such assessment and to ensure fair comparison of rates across studies, the final selection of studies had to include studies that used the same definition of readmission. To obtain the largest possible volume of previous research, the studies that used the same and most common definitions were included for the assessment of frequency. Furthermore, in order to be comparable to the Stockholm cohort described below, the sample had to include only older adults (age limit set to 60 years and/or specialized geriatric care) and without any additionally specified cohort criteria (eg, cohorts based on a particular diagnosis or a surgical intervention).

### Impact on Frequency of Readmission Based on Different Definitions

#### Setting and study cohort

The dataset used for illustration of how different criteria impact the risk of readmission, included a cohort of individuals discharged from geriatric inpatient care at any 1 of 3 publically run geriatric departments in the Stockholm region during 2016 and who lived in the region throughout the follow-up.

Data were extracted from the electronic health records at the geriatric departments. These were linked to data on subsequent healthcare on individual level, using a pseudonymized version of the Swedish national personal identification number. Informed consent was not collected due to the design of the study. The study was approved by the proper ethical review board detailed under declarations below.

The care delivered at the 3 departments is supposed to be equivalent and is based on the Comprehensive Geriatric Assessment (CGA),^22^ although no study-specific alignment of care processes has been performed. The study population has previously been presented in detail.^18,19^

#### Quantification and descriptive analysis

To illustrate how set criteria may affect rates of readmission, estimations were performed with the most common criteria found in the narrative review. Readmission rates with different criteria were assessed, and some sensitivity analyses were performed, including analysis of transfers to other departments and readmission frequency during day 0 after discharge.

**Figure 1.**
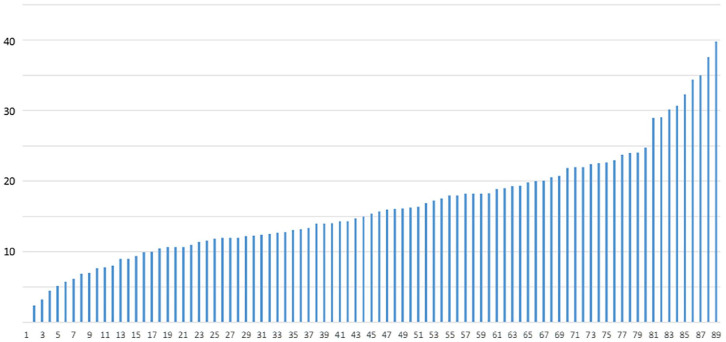
Readmission rates presented in the study selection; studies including all-cause, 30-day readmission rates. *y*-Axis denotes readmission frequency, and *x*-axis denotes different study cohorts. All studies are listed in the Supplemental Table S2. Out of these studies, the ones assessing several cohorts/subgroups contribute with 1 bar per cohort/subgroup.

Readmission of individuals previously admitted to a geriatric department included readmissions for any kind of reason at any department, as the purpose was to provide a comprehensive picture. Readmission rates found in the review were presented for selected studies individually to enable comparison (Figure 1), and presented as average and interquartile range (IQR, ie, the difference between quartiles 3 and 1) for the different categories of definitions (Table 2).

## Results

### Narrative Review

#### Definitions of readmission

Definitions of readmission that were identified within the selected studies included time-based criteria, either alone or combined with additional criteria, and criteria based on medical condition or on readmitting department. The rationale for selecting specific criteria were generally not reported if the study did not concern specific conditions or interventions.

In the selection for qualitative synthesis (Supplemental Figure S1), a defined time interval was the main criteria (95%, 265 out of 279 studies) for being considered a readmission. There were several examples of defining a readmission as after a particular intervention or after care for a specific diagnosis, most often combined with a time interval. The most common diagnosis was hip fracture (32 studies, 11% of studies included based on abstract screening). In 9 studies the definition of readmission was a combination of time delimiter and an additional criteria such as a specific diagnosis or diagnosis group for the event of readmission, for example, fall-related^23^ or stroke-related.^24^ Potentially avoidable readmissions were assessed in 5 studies, and this was done in addition to analyzing the overall readmission rate. An additional method to define what to consider a readmission was the same-diagnosis criteria, that is, a readmission had the same main diagnosis or belonged to the same diagnosis category in terms of ICD-10 sub-chapter (eg, stroke-related or psychiatric) as the index admission (in 7 studies, or 3%). Readmission based on a same-department criterion was explicitly used in 3 studies, all concerning emergency department.

Table 1 includes studies selected for the qualitative synthesis (selection process in Supplemental Figure S1). Only the most common time delimiters are presented.

**Table 1.** Most Common Readmission Definitions Identified in the Narrative Review.

Category of criteria for defining a readmission	Number of studies
Time delimiter only^a^	
14 days	4
30 days	155
90 days	44
180 days	28
365 days	32
Combination of time delimiter and additional criteria	9
Department dependent	3
Condition-based (with or without time delimiter)	
Same main diagnosis or category	7
Potentially avoidable	5

a Where the time delimiter was expressed in months instead of days, 1 month was translated to 30 days, 6 months to 180 days, and 12 months to 365 days.

#### Frequency of readmission

Studies with the most common definition were selected for assessment of readmission rates. Sixty-six studies (out of the 279 abstracts screened) analyzed readmission rates based on a 30-day time limit and regarding all causes of readmission for older adults; another almost 90 studies used the same time horizon but with additional criteria. Figure 1 presents readmission rates reported in each study. The studies found at the lower and higher ends of the scale did not offer obvious differences in terms of the methods or subjects, although their readmission rates ranged from 0% in 1 sub-cohort^25^ to above 30%.^26-28^

### Impact on Frequency of Readmission Based on Different Definitions

The cohort consisted of 8071 patients who were all admitted to 1 of 3 geriatric departments in Stockholm run by the Stockholm County council. Sixty-three percent were women, and the average age at index admission was 83.5 years. The cohort has been extensively described in previous studies.^18,19^

Based on findings from the narrative review, readmission rates were estimated (Table 2). The 30-day horizon was the most common time limit, why this was used when the readmission definition was expanded to specific variants, for example, based also on condition. Readmission rates from a selection of studies included in the narrative review were also reported, to enable comparison.

**Table 2.** Readmission Rates for the Swedish Cohort Discharged From Geriatric Inpatient Care. Includes Estimates From Studies Included in the Review; Department-dependent and Condition-based Readmission Were Not All Part of the Final Review Selection of 66 Studies.

Category of criteria for defining a readmission	Readmission rates in reviewed studies, median (IQR)	Implied readmission rate Stockholm cohort	Comment
Time delimiter only			
14 days		8.0%	
30 days	15.7% (8.7%)	12.6%	Based on all separate sub-cohorts
90 days		23.2%	
180 days		33.6%	
Department dependent (30-day time limit)			
Geriatric department		1.2%	No study assessed readmission at the same geriatric department^a^
Same department	12.5% (3.2%)	3.1% (0.2%)	Included all departments at the same unit^b^
Condition-based (30-day time limit)			
Same main diagnosis or category	6.1%	11.1%	Three-digit ICD-10 level
Potentially avoidable	3.5% (1.9%)		
Heart failure and hypertension		1.2%	ICD-10 codes I50^c^, I11^c^, I12^c^, I13^c^, I15^c^
Respiratory exacerbation/Pneumonia		1.4%	ICD-10 codes J10^c^ -J17^c^, J44^c^, J45^c^, J46^c^, J69^c^
Urinary tract condition		0.4%	ICD-10 codes N30^c^, N39^c^

Abbreviation: IQR, Interquartile range.

That is the difference between quartiles 3 and 1.

a The same-geriatric department criterion implied readmission at the same geriatric department as during the index admission. Such figures were not reported by studies included in the review but possible to calculate for the Stockholm cohort.

b The same-department criterion included all departments at a given unit, that is, not only geriatric departments.

Using longer time intervals for computing readmission rates had substantial effects on the outcome; a 14-day time interval implied a rate of 8.0% whilst a 30-day interval rendered a rate of 12.6%. The density of readmissions per day was higher during the first weeks after discharge and then dropped continuously. Excluding new admissions dated the same day as the day of discharge from the index admission implied a drop to 6.7% for the 14-day readmission rate and to 11.6% for the 30-day readmission rate. Including all registered admissions after discharge and hence not considering whether they were registered as transfers between departments implied a readmission rate of 9.5% (14 days) and 13.8% (30 days) respectively.

Readmission frequencies based on diagnosis (within the 30-day interval) differed between previous research identified in the narrative review and the Stockholm cohort; considering a new admission as a readmission only if it belonged to the same diagnosis category as the index event implied a twofold difference (6.1% versus 11.1%). As the definition of potentially avoidable readmission varied substantially in previous studies, a few examples of these were applied to the Stockholm cohort, with varying results (0.4%-1.4% versus 3.5%).

## Discussion

Readmission is a frequently used concept, and the use of it is often linked to quality of care, where quality of care could refer to the initial inpatient stay as well as to the outpatient care delivered by the primary care provider and/or other social care providers including, for example, the individual’s residential care facility. The present study aimed at putting the concept of readmission into context and assess its deployment and definitions. We found that time limits constituted the most common category of readmission definitions, with a 30-day span being the most common one—however, this was not explicitly motivated in a medical perspective in a single study. Furthermore, we found that for studies leveraging the same definitions on similar cohorts, the frequencies reported varied. Our application of different criteria to a cohort of older adults discharged from inpatient geriatric care in Stockholm showed that slight changes in, for example, time intervals impacted the frequency of readmission substantially. Hence, transparent reporting of criteria is important, to enable adequate judgement of the findings and to enable fair comparisons with other studies.

### Definitions of Readmission

Tightly linked to discussing definitions is the question of purpose with computing readmission, and the review of literature makes it clear that different definitions may be appropriate depending on context and what the results are used for. At the macro level, readmission is undoubtedly a useful measure understanding changes over time as well as differences between cohorts, if the definitions are genuinely harmonized. For the individual, readmission risk is relatively intangible. However, with transparent reporting and symmetric information, the individual could gain awareness of the performance for different caregiving units. For caregiving units, such as geriatric departments as in this case, it may be useful as a quality measure given that comparisons are done on harmonized definitions. Without harmonization of definitions, though, comparisons are useless.

Assessing avoidable readmission is an additional potential opportunity, to enable strategies to reduce these. This has been done in several studies, although it is debatable what to consider avoidable. It has been estimated in France that hospital readmissions for seniors above 75 years are to 25% avoidable.^29^

### Frequency of Readmission

Identified studies showed a span of average 30-day readmissions from 0% to more than 30%. Even though it may be precarious to compare readmission frequency for 2 different contexts, monitoring over time, that is, in the same context, could still be considered relevant. One excellent example of such comparison, is in evaluation of something new; for example, studying a new intervention^30^ or a different way of working,^31^ if possible within a randomized clinical trial or a retrospective case-control study.

### Readmission Rates When Different Criteria Are Applied

The Stockholm case example showed that frequency of readmission differed substantially depending on presumably small changes in criteria. Estimating all-cause readmission within the first 14 days after discharge implied a rate of 8.0% and extending the time horizon to 30 days implied a readmission rate of 12.6%.

There were similarities regarding frequency of readmission between the sample of reviewed studies and the Stockholm cohort (30-day readmission amounted to 14.0% versus 12.6% respectively) but also substantial differences (same-department readmission of 12.5% versus 3.1% respectively). The latter are likely due to that these publicly run departments managing geriatric patients in Stockholm were to a greater extent stand-alone and not part of major emergency hospitals as seemed to be the case in many of the reviewed studies.

### Strengths and Limitations

Limitations of the narrative review include the fact that the selection of adequate studies may be incomplete. To reduce this risk, expertise from the university library was used, and validation of the selections made was done within the research group. Strengths include transparent reporting of the studies identified and their features.

The composition of the study population is a limitation regarding generalizability of results from the cohort analysis; specialized geriatric inpatient care is not offered everywhere in the country nor in the world and the criteria for accessing geriatric inpatient care may vary over time as well as between contexts. The generalizability of findings from the cohort analysis is difficult to detail as the contextual factors are hard to disentangle, which is also an inherent challenge regarding generalization between contexts. The findings from the cohort analysis should however be generalizable for the administrative region, as validated in a previous study.^17^ Good data availability for the analyzed cohort enabled complete follow-up.

Implications on readmission risk are substantial with alterations of criteria no matter what characterizes the underlying patient population; time limits and other specifications as part of the readmission definitions have substantial impact and should be treated with caution when performing comparative analysis.

### Future Research

To enable better understanding of readmission over time, scientific reviews should be done on a regular basis. However, to understand what groups that would benefit from interventions to reduce readmission, research aimed at clusters with high degree of readmission is essential. Regardless, for lasting improvements in ways of working, there is a need for continuous monitoring and evaluation that do not necessarily have to be labeled as research.

## Conclusions

There are a few different categories of readmission definitions within research literature on older adults, of which time limit is the most common one. Frequency of readmission varied significantly between studies and settings for this heterogeneous group, pointing to that it is likely highly related to the organization of the healthcare system, including efforts before and immediately following discharge. Applying different definitions from a narrative review on a cohort of older adults in a healthcare system of universal healthcare, revealed that the design of definition has substantial impact on frequency of readmission.

Readmission rates are highly dependent on the study population and its context, and the actual use of readmission monitoring is dependent on what purpose it is supposed to fulfill. Specified time limits leveraged for readmission estimates are not primarily based on medical reasoning, but economic and structural. To enable practical use of measuring readmission, it is important to assess them in the context of all relevant stakeholders including the inpatient care facility, primary care providers and social care providers throughout the community.

## Supplemental Material

sj-docx-1-jpc-10.1177_21501319251320181 – Supplemental material for Readmission After Geriatric Inpatient Care: A Narrative Review and a Comparative Analysis Supplemental material, sj-docx-1-jpc-10.1177_21501319251320181 for Readmission After Geriatric Inpatient Care: A Narrative Review and a Comparative Analysis by Carl Willers, Rikard Lindqvist, Martin Dreilich, Stefan Fors, Amelie Lindh Mazya, Gunnar H. Nilsson, Anne-Marie Boström, Mahwish Naseer and Elisabeth Rydwik in Journal of Primary Care & Community Health

sj-png-2-jpc-10.1177_21501319251320181 – Supplemental material for Readmission After Geriatric Inpatient Care: A Narrative Review and a Comparative Analysis Supplemental material, sj-png-2-jpc-10.1177_21501319251320181 for Readmission After Geriatric Inpatient Care: A Narrative Review and a Comparative Analysis by Carl Willers, Rikard Lindqvist, Martin Dreilich, Stefan Fors, Amelie Lindh Mazya, Gunnar H. Nilsson, Anne-Marie Boström, Mahwish Naseer and Elisabeth Rydwik in Journal of Primary Care & Community Health
