# Supplementary material for: Readmission After Geriatric Inpatient Care: A Narrative Review and a Comparative Analysis
Source: J Prim Care Community Health. 2025 Feb 27;16:21501319251320181. doi: 10.1177/21501319251320181 (PMC11869310; doi:10.1177/21501319251320181)
Supplement: sj-docx-1-jpc-10.1177_21501319251320181 – Supplemental material for Readmission After Geriatric Inpatient Care: A Narrative Review and a Comparative Analysis [file sj-docx-1-jpc-10.1177_21501319251320181.docx]

**Supplementary material**

**Table S1**. Search query in literature review; a) PubMed, b) Embase

**Table S2**. Studies included in final selection of narrative review and introduced in Figure 1 (selection specified in Figure S1).

Bellon, J. E., Bilderback, A., Ahuja-Yende, N. S., Wilson, C., Altieri Dunn, S. C., Brodine, D., & Boninger, M. L. (2019). University of Pittsburgh Medical Center Home Transitions Multidisciplinary Care Coordination Reduces Readmissions for Older Adults. Journal of the American Geriatrics Society, 67(1), 156–163. https://doi.org/10.1111/jgs.15643

Bernstein, J. M., Graven, P., Drago, K., Dobbertin, K., & Eckstrom, E. (2018). Higher Quality, Lower Cost with an Innovative Geriatrics Consultation Service. Journal of the American Geriatrics Society, 66(9), 1790–1795. https://doi.org/10.1111/jgs.15473

Bogaisky, M., & Dezieck, L. (2015). Early hospital readmission of nursing home residents and community-dwelling elderly adults discharged from the geriatrics service of an urban teaching hospital: patterns and risk factors. Journal of the American Geriatrics Society, 63(3), 548–552. https://doi.org/10.1111/jgs.13317

Chiu, P., Lee, A., See, T., & Chan, F. (2018). Outcomes of a pharmacist-led medication review programme for hospitalised elderly patients. Hong Kong Medical Journal = Xianggang Yi Xue Za Zhi, 24(2), 98–106. <https://doi.org/10.12809/hkmj176871>

Cramon, M. Ø., et al. (2021). "Individual nutritional intervention for prevention of readmission among geriatric patients—a randomized controlled pilot trial." Pilot and Feasibility Studies 7(1).

Dedhia, P., Kravet, S., Bulger, J., Hinson, T., Sridharan, A., Kolodner, K., Wright, S., & Howell, E. (2009). A quality improvement intervention to facilitate the transition of older adults from three hospitals back to their homes. Journal of the American Geriatrics Society, 57(9), 1540–1546. https://doi.org/10.1111/j.1532-5415.2009.02430.x

Dinescu, A., Korc-Grodzicki, B., Farber, J., & Ross, J. S. (2012). Discharge disposition disagreements and re-admission risk among older adults: a retrospective cohort study. BMJ Open, 2(6). <https://doi.org/10.1136/bmjopen-2012-001646>

Dramé M, Hombert V, Cantegrit E, Proye E, Godaert L. Derivation and validation of a 90-day unplanned hospital readmission score in  older patients discharged form a geriatric ward. Eur Geriatr Med. 2022 Oct;13(5):1119–25.

Dresden, S. M., Hwang, U., Garrido, M. M., Sze, J., Kang, R., Vargas-Torres, C., Courtney, D. M., Loo, G., Rosenberg, M., & Richardson, L. (2020). Geriatric Emergency Department Innovations: The Impact of Transitional Care Nurses on 30-day Readmissions for Older Adults. Academic Emergency Medicine : Official Journal of the Society for Academic Emergency Medicine, 27(1), 43–53. https://doi.org/10.1111/acem.13880

Eriksen AV, Thrane MD, Matzen L, Ryg J, Andersen-Ranberg K. Older patients acutely admitted and readmitted to the same geriatric department:  a descriptive cohort study of primary diagnoses and health characteristics. Eur Geriatr Med. 2022 Oct;13(5):1109–18.

Farhat, N. M., Vordenberg, S. E., Marshall, V. D., Suh, T. T., & Remington, T. L. (2019). Evolution of interdisciplinary geriatric transitions of care on readmission rates. The American Journal of Managed Care, 25(7), e219–e223.

Farlie MK, French F, Haines TP, Taylor NF, Williams C. The impact of additional allied health staffing on rehabilitation outcomes at discharge from a sub-acute geriatric evaluation and management unit: A quasi-experimental, pre-post intervention study. Clin Rehabil. 2022 Aug;36(8):1110–9.

Fitriana I, Setiati S, Rizal EW, Istanti R, Rinaldi I, Kojima T, et al. Malnutrition and depression as predictors for 30-day unplanned readmission in older patient: a prospective cohort study to develop 7-point scoring system. BMC Geriatr. 2021 Apr;21(1):256.

Gregersen M, Hansen TK, Jørgensen BB, Damsgaard EM. Frailty is associated with hospital readmission in geriatric patients: a prognostic study. Eur Geriatr Med. 2020 May;

Han SJ, Jung H-W, Lee JH, Lim J, Moon S do, Yoon S-W, et al. Clinical Frailty Scale, K-FRAIL questionnaire, and clinical outcomes in an acute hospitalist unit in Korea. Korean J Intern Med. 2021 Sep;36(5):1233–41.

Hansen TK, Pedersen LH, Shahla S, Damsgaard EM, Bruun JM, Gregersen M. Effects of a new early municipality-based versus a geriatric team-based transitional care intervention on readmission and mortality among frail older patients - a randomised controlled trial. Arch Gerontol Geriatr. 2021;97:104511.

Hellemans L, Hias J, Walgraeve K, Flamaing J, Spriet I, Tournoy J, et al. Deprescribing in geriatric inpatients is associated with a lower readmission risk: a case control study. Int J Clin Pharm. 2020 Oct;42(5):1374–8.

Huckfeldt, P. J., Reyes, B., Engstrom, G., Yang, Q., Diaz, S., Fahmy, S., & Ouslander, J. G. (2019). Evaluation of a Multicomponent Care Transitions Program for High-Risk Hospitalized Older Adults. Journal of the American Geriatrics Society, 67(12), 2634–2642. https://doi.org/10.1111/jgs.16189

Iloabuchi, T. C., Mi, D., Tu, W., & Counsell, S. R. (2014). Risk factors for early hospital readmission in low-income elderly adults. Journal of the American Geriatrics Society, 62(3), 489–494. https://doi.org/10.1111/jgs.12688

Jiao J, Guo N, Xie L, Ying Q, Zhu C, Guo X, et al. Association between Frailty and 90-Day Outcomes amongst the Chinese Population: A Hospital-Based Multicentre Cohort Study. Gerontology. 2022;68(1):8–16.

Kerminen HM, Jäntti PO, Valvanne JNA, Huhtala HSA, Jämsen ERK. Risk factors of readmission after geriatric hospital care: An interRAI-based cohort study in Finland. Arch Gerontol Geriatr. 2021;94:104350.

Keyes, D. C., Singal, B., Kropf, C. W., & Fisk, A. (2014). Impact of a new senior emergency department on emergency department recidivism, rate of hospital admission, and hospital length of stay. Annals of Emergency Medicine, 63(5), 517–524. https://doi.org/10.1016/j.annemergmed.2013.10.033

Klinge, M., Aasbrenn, M., Öztürk, B., Christiansen, C. F., Suetta, C., Pressel, E., & Nielsen, F. E. (2020). Readmission of older acutely admitted medical patients after short-term admissions in Denmark: a nationwide cohort study. BMC Geriatrics, 20(1), 203. https://doi.org/10.1186/s12877-020-01599-4

Kongensgaard R, Hansen TK, Krogseth M, Gregersen M. Impact of involvement of relatives in early home visits by a hospital-led geriatric team. Geriatr Nurs. 2022;45:64–8.

Krol, M. L., Allen, C., Matters, L., Jolly Graham, A., English, W., & White, H. K. (2019). Health Optimization Program for Elders: Improving the Transition From Hospital to Skilled Nursing Facility. Journal of Nursing Care Quality, 34(3), 217–222. <https://doi.org/10.1097/NCQ.0000000000000375>

Laura T, Melvin C, Yoong DY. Depressive symptoms and malnutrition are associated with other geriatric syndromes and increase risk for 30-Day readmission in hospitalized older adults: a prospective cohort study. BMC Geriatr. 2022 Aug;22(1):634.

Lee JY, Kim KJ, Choi JW, Kim TH, Kim CO. Factors Related to Hospital Readmission of Frail Older Adults in Korea. Yonsei Med J. 2022 Nov;63(11):984–90.

Leite HT, Manhães AC, Antunes LA, Chan T, Hajj-Boutros G, Morais JA. The Implementation of a Geriatrics Co-Management Model of Care Reduces Hospital Length of Stay. Healthc (Basel, Switzerland). 2022 Oct;10(11).

Lin C-F, Huang Y-H, Ju L-Y, Weng S-C, Lee Y-S, Chou Y-Y, et al. Health-Related Quality of Life Measured by EQ-5D in Relation to Hospital Stay and Readmission in Elderly Patients Hospitalized for Acute Illness. Int J Environ Res Public Health. 2020 Jul;17(15).

Lin, K.-P., Chen, J.-H., Lu, F.-P., Wen, C.-J., & Chan, D.-C. D. (2019). The impact of early comprehensive geriatric screening on the readmission rate in an acute geriatric ward: a quasi-experimental study. BMC Geriatrics, 19(1), 285. <https://doi.org/10.1186/s12877-019-1312-y>

Mak JKL, Hägg S, Eriksdotter M, Annetorp M, Kuja-Halkola R, Kananen L, et al. Development of an Electronic Frailty Index for Hospitalized Older Adults in Sweden. J Gerontol A Biol Sci Med Sci. 2022 Nov;77(11):2311–9.

McCusker J, Warburton RN, Lambert SD, Belzile E, de Raad M. The Revised Identification of Seniors At Risk screening tool predicts readmission in older hospitalized patients: a cohort study. BMC Geriatr. 2022 Nov;22(1):888.

McGrath, J., Almeida, P., & Law, R. (2019). The Whittington Frailty Pathway: improving access to comprehensive geriatric assessment: an interdisciplinary quality improvement project. BMJ Open Quality, 8(4), e000798. https://doi.org/10.1136/bmjoq-2019-000798

Melgaard, D., Rodrigo-Domingo, M., Mørch, M. M., & Byrgesen, S. M. (2019). DEMMI Scores, Length of Stay, and 30-Day Readmission of Acute Geriatric Patients in Denmark: A Cross-Sectional Observational Study with Longitudinal Follow-Up. Geriatrics (Basel, Switzerland), 4(1). https://doi.org/10.3390/geriatrics4010008

Mixon, A. S., Yeh, V. M., Simmons, S., Powers, J., Ely, E. W., Schnelle, J., & Vasilevskis, E. E. (2019). Improving Care Transitions for Hospitalized Veterans Discharged to Skilled Nursing Facilities: A Focus on Polypharmacy and Geriatric Syndromes. Geriatrics (Basel, Switzerland), 4(1). https://doi.org/10.3390/geriatrics4010019

Munjal, K. G., Shastry, S., Chapin, H., Tan, N., Misra, A., Greenberg, E., Traisman, B., Kleiman, R., Loo, G., Grudzen, C., Chason, K., & Richardson, L. D. (2020). Retrospective Cohort Study of Rates of Return Emergency Department Visits Among Patients Transported Home by Ambulance. The Journal of Emergency Medicine. https://doi.org/10.1016/j.jemermed.2020.04.043

Oates, D. J., Kornetsky, D., Winter, M. R., Silliman, R. A., Caruso, L. B., Sharbaugh, M. E., Hardt, E. J., & Parker, V. A. (2013). Minimizing geriatric rehospitalizations: a successful model. American Journal of Medical Quality : The Official Journal of the American College of Medical Quality, 28(1), 8–15. <https://doi.org/10.1177/1062860612445181>

Ohta R, Sano C. Risk of Hospital Readmission among Older Patients Discharged from the Rehabilitation Unit in a Rural Community Hospital: A Retrospective Cohort Study. J Clin Med. 2021 Feb;10(4).

Ouslander, J. G., Reyes, B., Diaz, S., & Engstrom, G. (2020). Thirty-Day Hospital Readmissions in a Care Transitions Program for High-Risk Older Adults. Journal of the American Geriatrics Society, 68(6), 1307–1312. https://doi.org/10.1111/jgs.16314

Parsons, P. L., & Gifford, C. (2002). Characteristics and outcomes of elderly patients receiving transitional care. Outcomes Management, 6(4), 182–185.

Petigara, S., Krishnamurthy, M., & Livert, D. (2017). Necessity is the mother of invention: an innovative hospitalist-resident initiative for improving quality and reducing readmissions from skilled nursing facilities. Journal of Community Hospital Internal Medicine Perspectives, 7(2), 66–69. <https://doi.org/10.1080/20009666.2017.1313492>

Pereira F, Verloo H, Zhivko T, Di Giovanni S, Meyer-Massetti C, von Gunten A, et al. Risk of 30-day hospital readmission associated with medical conditions and drug regimens of polymedicated, older inpatients discharged home: a registry-based cohort study. BMJ Open. 2021 Jul;11(7):e052755.

Poulsen SH, Rosenvinge PM, Modlinski RM, Olesen MD, Rasmussen HH, Holst M. Signs of dysphagia and associated outcomes regarding mortality, length of hospital stay and readmissions in acute geriatric patients: Observational prospective study. Clin Nutr ESPEN. 2021 Oct;45:412–9.

Powers JS, Abraham L, Parker R, Azubike N, Habermann R. The GeriPACT Initiative to Prevent All-Cause 30-Day Readmission in High Risk Elderly. Geriatr (Basel, Switzerland). 2021 Jan;6(1).

Pugh, J. A., Wang, C.-P., Espinoza, S. E., Noël, P. H., Bollinger, M., Amuan, M., Finley, E., & Pugh, M. J. (2014). Influence of frailty-related diagnoses, high-risk prescribing in elderly adults, and primary care use on readmissions in fewer than 30 days for veterans aged 65 and older. Journal of the American Geriatrics Society, 62(2), 291–298. <https://doi.org/10.1111/jgs.12656>

Ramsey KA, Rojer AGM, van Garderen E, Struik Y, Kay JE, Lim WK, et al. The Association of Changes in Physical Performance During Geriatric Inpatient Rehabilitation With Short-Term Hospital Readmission, Institutionalization, and Mortality: RESORT. J Am Med Dir Assoc. 2022 Nov;23(11):1883.e1-1883.e8.

Rosted, E., Poulsen, I., Hendriksen, C., Petersen, J., & Wagner, L. (2013). Testing a two step nursing intervention focused on decreasing rehospitalizations and nursing home admission post discharge from acute care. Geriatric Nursing (New York, N.Y.), 34(6), 477–485. <https://doi.org/10.1016/j.gerinurse.2013.08.001>

Samuel S V, Viggeswarpu S, Wilson BP, Ganesan MP. Readmission rates and predictors of avoidable readmissions in older adults in a tertiary care centre. J Fam Med Prim care. 2022 Sep;11(9):5246–53.

Schapira M, Outumuro MB, Giber F, Pino C, Mattiussi M, Montero-Odasso M, et al. Geriatric co-management and interdisciplinary transitional care reduced hospital readmissions in frail older patients in Argentina: results from a randomized controlled trial. Aging Clin Exp Res. 2022 Jan;34(1):85–93.

Schiltz NK, Dolansky MA, Warner DF, Stange KC, Gravenstein S, Koroukian SM. Impact of Instrumental Activities of Daily Living Limitations on Hospital Readmission: an Observational Study Using Machine Learning. J Gen Intern Med. 2020 Oct;35(10):2865–72.

Segelman, M., Szydlowski, J., Kinosian, B., McNabney, M., Raziano, D. B., Eng, C., van Reenen, C., & Temkin-Greener, H. (2014). Hospitalizations in the Program of All-Inclusive Care for the Elderly. Journal of the American Geriatrics Society, 62(2), 320–324. https://doi.org/10.1111/jgs.12637

Shebehe, J., & Hansson, A. (2018). High hospital readmission rates for patients aged ≥65 years associated with low socioeconomic status in a Swedish region: a cross-sectional study in primary care. Scandinavian Journal of Primary Health Care, 36(3), 300–307. https://doi.org/10.1080/02813432.2018.1499584

Shen Y, Hao Q, Liu S, Su L, Sun X, Flaherty JH, et al. The impact of functional status on LOS and readmission in older patients in geriatrics department: a cohort study. Aging Clin Exp Res. 2020 Oct;32(10):1977–83.

Shin, J., et al. (2020). "Importance of geriatric syndrome screening within 48 hours of hospitalization for identifying readmission risk: A retrospective study in an acute-care hospital." Annals of Geriatric Medicine and Research 24(2): 83-90.

Sinvani, L., Carney, M., Kozikowski, A., Smilios, C., Patel, V., Qiu, G., Zhang, M., Babalola, O., Kandov, Y., Rosenberg, D., Wolf-Klein, G., & Pekmezaris, R. (2018). The role of geriatrician-hospitalists in the care of older adults: A retrospective cohort study. Archives of Gerontology and Geriatrics, 77, 31–37. <https://doi.org/10.1016/j.archger.2018.03.006>

Solakoglu, G. A., et al. (2021). "Can frailty tools predict the mortality, readmission, and hospitalization of geriatric emergency attenders?" International Journal of Gerontology 15(2): 145-149.

Strait, L. A., Fitzgerald, E., Zurmehly, J., & Overcash, J. (2019). A Congregation Transition of Care Program Using Faith Community Nurses and Volunteer Faith-Based Nurses. Journal of Christian Nursing : A Quarterly Publication of Nurses Christian Fellowship, 36(3), 158–165. <https://doi.org/10.1097/CNJ.0000000000000625>

Sun C-H, Chou Y-Y, Lee Y-S, Weng S-C, Lin C-F, Kuo F-H, et al. Prediction of 30-Day Readmission in Hospitalized Older Adults Using Comprehensive Geriatric Assessment and LACE Index and HOSPITAL Score. Int J Environ Res Public Health. 2022 Dec;20(1).

Takahashi, P. Y., Haas, L. R., Quigg, S. M., Croghan, I. T., Naessens, J. M., Shah, N. D., & Hanson, G. J. (2013). 30-day hospital readmission of older adults using care transitions after hospitalization: a pilot prospective cohort study. Clinical Interventions in Aging, 8, 729–736. https://doi.org/10.2147/CIA.S44390

Takahashi, P. Y., Naessens, J. M., Peterson, S. M., Rahman, P. A., Shah, N. D., Finnie, D. M., Weymiller, A. J., Thorsteinsdottir, B., & Hanson, G. J. (2016). Short-term and long-term effectiveness of a post-hospital care transitions program in an older, medically complex population. Healthcare (Amsterdam, Netherlands), 4(1), 30–35. <https://doi.org/10.1016/j.hjdsi.2015.06.006>

Thomsen K, Fournaise A, Matzen LE, Andersen-Ranberg K, Ryg J. Does geriatric follow-up visits reduce hospital readmission among older patients discharged to temporary care at a skilled nursing facility: a before-and-after cohort study. BMJ Open. 2021 Aug;11(8):e046698.

Umegaki H, Nagae M, Komiya H, Watanabe K, Yamada Y, Sakai T, et al. Clinical significance of geriatric conditions in acute hospitalization. Geriatr Gerontol Int. 2023 Jan;23(1):50–3.

Visade F, Babykina G, Puisieux F, Bloch F, Charpentier A, Delecluse C, et al. Risk Factors for Hospital Readmission and Death After Discharge of Older Adults from Acute Geriatric Units: Taking the Rank of Admission into Account. Clin Interv Aging. 2021;16:1931–41.

Wan CS, Reijnierse EM, Maier AB. Risk Factors of Readmissions in Geriatric Rehabilitation Patients: RESORT. Arch Phys Med Rehabil. 2021 Aug;102(8):1524–32.

Wang, P., Wang, Q., Li, F., Bian, M., & Yang, K. (2019). Relationship Between Potentially Inappropriate Medications And The Risk Of Hospital Readmission And Death In Hospitalized Older Patients. Clinical Interventions in Aging, 14, 1871–1878. https://doi.org/10.2147/CIA.S218849

Wilson, H., Loke, Y. K., Hamilton, E. J., Green, A. Q., Southgate, J. L., Markham, E., & Chomicki, P. (2014). Readmission rates of older patients (age >75 years) discharged within 48 hours of admission to the Acute Medical Unit, Norwich: observational study. Future Hospital Journal, 1(1), 23–25. <https://doi.org/10.7861/futurehosp.14.008>


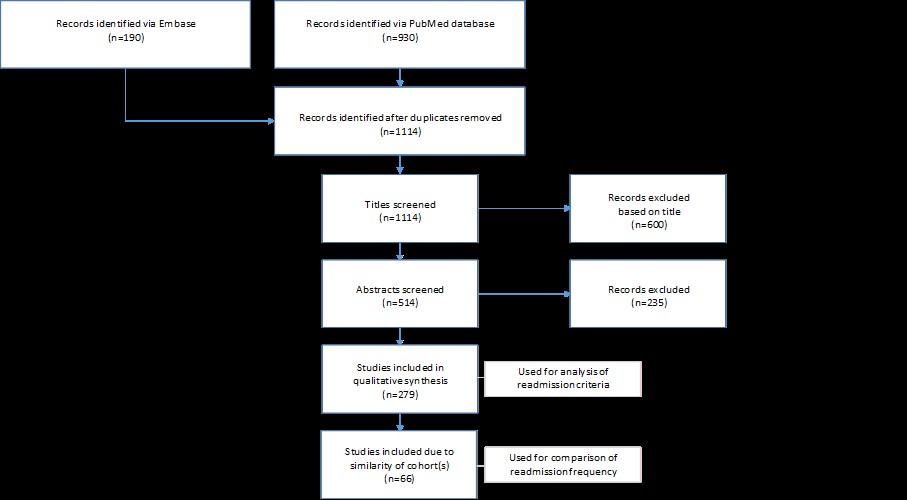


**Figure S1**. PRISMA flow diagram for the literature review process
