# Supplementary figures and images for: Readmission After Geriatric Inpatient Care: A Narrative Review and a Comparative Analysis
Source: J Prim Care Community Health. 2025 Feb 27;16:21501319251320181. doi: 10.1177/21501319251320181 (PMC11869310; doi:10.1177/21501319251320181)

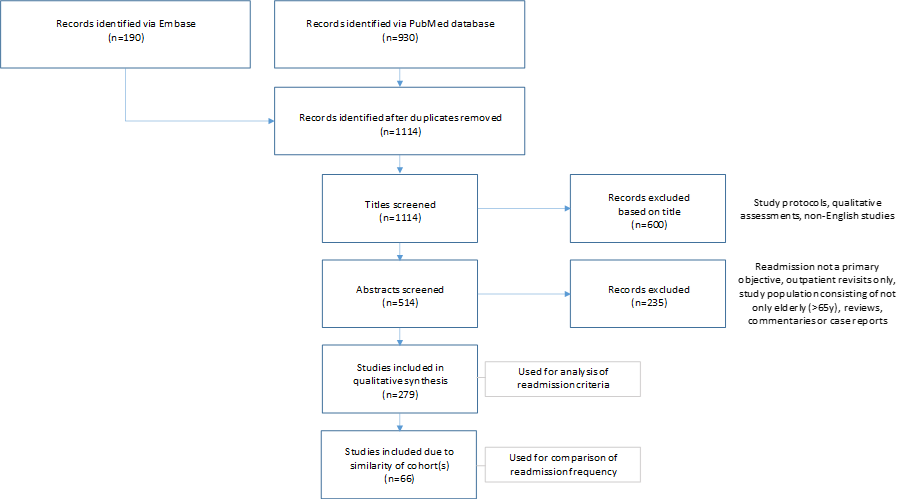

Supplement: sj-png-2-jpc-10.1177_21501319251320181 – Supplemental material for Readmission After Geriatric Inpatient Care: A Narrative Review and a Comparative Analysis [file sj-png-2-jpc-10.1177_21501319251320181.png]
